# Quantifying Child-Appeal: The Development and Mixed-Methods Validation of a Methodology for Evaluating Child-Appealing Marketing on Product Packaging

**DOI:** 10.3390/ijerph18094769

**Published:** 2021-04-29

**Authors:** Christine Mulligan, Monique Potvin Kent, Laura Vergeer, Anthea K. Christoforou, Mary R. L’Abbé

**Affiliations:** 1 Department of Nutritional Sciences, University of Toronto, Toronto, ON M5S 1A8, Canada; christine.mulligan@mail.utoronto.ca (C.M.); laura.vergeer@mail.utoronto.ca (L.V.); anthea.christoforou@mail.utoronto.ca (A.K.C.); 2 School of Epidemiology and Public Health, Faculty of Medicine, University of Ottawa, Ottawa, ON K1H 8M5, Canada; monique.potvinkent@uottawa.ca

**Keywords:** child-appealing marketing, marketing to kids, food marketing, marketing power, marketing techniques, validation, mixed methods, product packaging, food packaging

## Abstract

There is no standardized or validated definition or measure of “child-appeal” used in food and beverage marketing policy or research, which can result in heterogeneous outcomes. Therefore, this pilot study aimed to develop and validate the child-appealing packaging (CAP) coding tool, which measures the presence, type, and power of child-appealing marketing on food packaging based on the marketing techniques displayed. Children (n = 15) participated in a mixed-methods validation study comprising a binary classification (child-appealing packaging? Yes/No) and ranking (order of preference/marketing power) activity using mock breakfast cereal packages (quantitative) and focus group discussions (qualitative). The percent agreement, Cohen’s Kappa statistic, Spearman’s Rank correlation, and cross-classification analyses tested the agreement between children’s and the CAP tool’s evaluation of packages’ child-appeal and marketing power (criterion validity) and the content analysis tested the relevance of the CAP marketing techniques (content validity). There was an 80% agreement, and “moderate” pairwise agreement (κ [95% CI]: 0.54 [0.35, 0.73]) between children/CAP binary classifications and “strong” correlation (*r_s_* [95% CI]: 0.78 [0.63, 0.89]) between children/CAP rankings of packages, with 71.1% of packages ranked in the exact agreement. The marketing techniques included in the CAP tool corresponded to those children found pertinent. Pilot results suggest the criterion/content validity of the CAP tool for measuring child-appealing marketing on packaging in accordance with children’s preferences.

## 1. Introduction

Child-appealing marketing for foods and beverages of poor nutritional quality is pervasive [1,2,3,4,5]. These marketing practices have been shown to influence children’s taste preferences, purchase requests, and consumption patterns [2,4,6]. As a result, child-appealing marketing is contributing to poor diet quality and the growing burden of childhood obesity and diet-related chronic disease [1,2,7]. In response, the World Health Organization has recommended limiting the exposure and power of child-appealing marketing as a preventative action against childhood obesity [8,9]. Many countries are considering or have already implemented mandatory or voluntary child-appealing marketing restrictions [10,11], and recent evidence points to the potential effectiveness of these policies in improving the healthfulness of the food supply and consequently, children’s health [12,13,14,15].

A critical consideration in this field is the definition of “child-appeal”. However, there is currently no standardized framework by which to determine whether instances of marketing are appealing to children (i.e., does it have characteristics that children notice and find salient, therefore increasing their preference for the product), which has resulted in considerable heterogeneity in both policy and research [16,17]. A recent review inventoried 117 unique marketing techniques that have been used in child-appealing research, finding significant variability in the number and nature of techniques used across publications [16]. This lack of consistency is problematic given the potential for differential outcomes or interpretations of results, which could in turn lead to differential (and potentially less effective) policies and policy outcomes. Furthermore, the review found that many of the marketing techniques that were most frequently used in research were techniques that would not typically be considered “child-appealing” or be included in regulatory definitions of child-appeal, for example, nutrition and health marketing (e.g., nutrition claims) [16]. These findings suggest the need to increase the breadth of operationalized definitions of child-appeal to include a wider range of marketing techniques. 

Additionally, while many studies have addressed the “exposure” or the extent of child-appealing marketing, few have made efforts to formally quantify its “power” or persuasiveness [17,18]. Evidence suggests that the frequency of marketing exposure and the number of marketing instances displayed on a food package increases the power of the marketing message [19,20], and could therefore influence the children’s attraction to these foods. The type of marketing techniques employed by manufacturers can also alter persuasiveness [17,21]. 

Moreover, despite the number of studies that have used methodologies or coding tools to measure child-appeal, few have been validated. One study has validated a measure of brand awareness among children [22], however, there have been no studies aiming to validate methodologies to assess child-appealing food marketing in terms of how well they measure the aspects of marketing that children actually find appealing. There has been one study that developed and validated a teen-informed coding tool [21], but this type of consumer-validated coding tool is lacking for the child demographic. 

Recent evidence has found that product packaging is one of the top sources of children’s exposure to child-appealing marketing, and that most exposures are for foods of poor nutritional quality [23]. Monitoring marketing activities in this medium may therefore be particularly important. However, as with most mediums, there is no standardized or validated methodology for this purpose and the concept of marketing power has not been clearly elucidated in this context [16,17]. Therefore, the objectives of this study were to: (1) Develop a coding tool to measure the presence, type, and power of child-appealing marketing on product packaging and (2) validate the coding tool using a mixed-methods study with children. 

## 2. Materials and Methods

### 2.1. Development of the Child-Appealing Packaging (CAP) Coding Tool

The child-appealing packaging (CAP) coding tool was developed as a novel methodology to measure the presence, type, and power of child-appealing marketing on product packaging, by evaluating the marketing techniques displayed on the package. The CAP tool was developed by selecting marketing techniques from a published inventory of marketing techniques that have previously been used in child-appealing marketing research [16]. The CAP tool includes marketing techniques that are popular on product packaging specifically (e.g., cartoon characters, toys in the box), as well as techniques that have traditionally been used in other marketing platforms but are now appearing on packaging with evolving marketing practices (e.g., social media handles, scannable codes linked to websites). 

Following the selection of marketing techniques, the included techniques were categorized into two categories: Core techniques and broad techniques. Core techniques are marketing techniques that could independently make a package appealing to children (e.g., cartoon characters or games on the package). Core techniques are also those that are typically included in marketing regulations or restrictions, as these are more “objectively” or defensibly appealing to children. Broad techniques are marketing techniques that would not on their own cause a product to be considered “child-appealing”, however, in addition to the core techniques, these could increase the power of the marketing message as a whole (e.g., appeals to nutrition, health or value). Evidence has shown that marketing techniques such as promoting a product’s health, nutritional or economic value were amongst the most popular techniques used in child-appealing marketing research, despite them not being typical child-appealing techniques [16,17]. Broad techniques also include marketing techniques that may not appeal directly to children but may appeal to their parents or caregivers (e.g., product benefit claims, convenient packaging), and therefore be purchased for children. These techniques are important to monitor, given that when/if child-appealing marketing is restricted, a proliferation of broad techniques may occur as a means for manufacturers to circumvent regulations and ensure that their products are still consumed by children.

The selection and categorization of marketing techniques for the CAP tool was an iterative process, reviewed and adjusted multiple times by the research team to ensure face validity (i.e., ensuring that the tool logically appears to measure child-appealing marketing) [24] and to ensure that marketing techniques were clearly defined and mutually exclusive.

There are three primary outcome variables of the application of the CAP tool related to (1) the presence, (2) the type, and (3) the power of child-appealing marketing, detailed in Table 1. Briefly, the CAP tool measures the presence of child-appealing marketing (i.e., if the package is child-appealing or not), based on the display of one or more core marketing technique(s), and captures the type of the marketing based on the presence or frequency of display of individual core/broad marketing techniques within the sample. Finally, the CAP tool scores marketing power by summing all the techniques (core and broad) displayed on the package, based on evidence that the number of marketing techniques displayed on the package increases the persuasiveness or intensity of the marketing message [19,20]. For the purposes of the CAP tool, the presence of each marketing technique is weighted equally (i.e., 1 point each).

### 2.2. Study Design

As with any newly developed tool or methodology, the validation of the CAP tool prior to its application was necessary to ensure that the CAP tool is accurately measuring child-appealing marketing on product packaging. Therefore, this study undertook a mixed methods approach to test the criterion and content validity of the CAP tool. The quantitative study was a cross-sectional survey study, involving a product packaging classification activity and the qualitative study used focus group discussions—both studies were completed by children. A mixed methods approach was employed due to its usefulness in generating complementary types of data to answer different aspects of a research question (e.g., different types of validity) [25]. The current study was a pilot study, with a larger primary study planned, pending the results of this work and the evolving social distancing and safety protocols in place due to the COVID-19 pandemic.

Criterion validity is defined as the “extent to which the method is accurately based on an externally derived gold standard and examines whether a method correlates in a predicted manner with variables with which, theoretically, it should correlate” [26,27]. In the context of this study, criterion validity was tested by assessing how well the CAP tool’s evaluation of child-appealing marketing correlates with how children perceive and respond to various marketing techniques on product packaging—children’s perceptions being the “gold standard”. Theoretically, a product that the CAP tool identifies as displaying child-appealing packaging should align with whether children think the product is appealing to them or meant for kids. Similarly, a product with higher child-appealing marketing power, as scored by the CAP tool, should be more appealing to children. 

Content validity is defined as the “extent to which the system covers the full range of meaning for the concept being measured” [26,28]. For the CAP tool to have high content validity, it should capture and measure the entire breadth of what “child-appealing packaging” entails. Given that the CAP tool was developed based on a published inventory of marketing techniques that are known to be relevant to child-appealing marketing on product packaging [16], it inherently has some content validity. However, to additionally test the content validity of the CAP tool, children were asked to describe the aspects of product packaging that are important to them and key to driving child-appeal (in their opinion), to ensure that all marketing techniques that children find meaningful and salient are included in the CAP tool. 

This study was registered as a clinical trial (#NCT04294121) and was approved by the University of Toronto Human Research Ethics board (Protocol ID 37436). The registered protocols include an element related to parents’ perspectives on packaging, however, this was delayed due to the COVID-19 pandemic. 

### 2.3. Participants and Recruitment

Children (n = 15) were recruited using posters and emails distributed through community-based after-school child-care programs in Toronto, Canada. Children were eligible to participate if they were aged 5–13 years, any gender, and spoke English. Recruitment was conducted in neighbourhoods of varying socioeconomic statuses (SES), identified by publicly available Toronto census data, and based on the prevalence of low-income-after-tax households [29].

All study sessions occurred at the University of Toronto Nutrition Intervention Centre. At the time of participation, a written consent was obtained from the children’s parents or guardians and a written assent was obtained from the children. Families were compensated with gift vouchers for grocery retailers. 

### 2.4. Design of Mock Cereal Packages

A set of six mock-branded breakfast cereal packages were professionally designed for use in this study by a graphic design company with expertise in commercial food label design. Mock packages were used to reduce response bias based on brand-familiarity or preference. The breakfast cereal was chosen as the example product type, given their frequent display of child-appealing marketing on packaging [30,31,32,33,34] and consumption by children [35,36]. All of the boxes displayed the same nutritional information (i.e., nutrition facts table and ingredients list) to reduce bias based on the nutrient or ingredient content. Six cereal boxes were designed to portray both core and broad marketing techniques with a range of marketing power scores, as would be measured using the CAP tool. According to the CAP tool, four out of six cereals would be considered to have child-appealing packaging (i.e., displaying at least 1 core technique) and marketing power scores would range from 0–14 points and could therefore be ranked from 1 to 6 based on marketing power. A full description of the mock cereal packages can be found in Supplementary Table S1.

### 2.5. Study Protocol

Fifteen children were recruited to participate in 4 study sessions, ranging from 2 to 6 children each, meeting recommendations for the ideal children’s focus group sizes [37,38,39]. The demographic information for participants is presented in Table 2. In one study session, each child individually completed a classification activity using the breakfast cereals, and then participated in a focus group discussion with all other children. During the individual classification activity, children were asked to observe all sides of the six cereal packages and were asked (1) to decide if each of the individual cereal boxes was for children (i.e., a binary (yes/no) response) and (2) to put the cereal boxes in order of which they liked the most to which they liked the least (i.e., a ranked (1 to 6) response). Children’s responses were recorded by a member of the research team. The children who were awaiting participation were kept occupied by another member of the research team in a separate area as to not bias their responses by hearing the previous children’s responses. Boxes were rearranged between participants into a predetermined random order to ensure consistency between participants.

Following the completion of the classification activity, children participated in the focus group discussion, moderated by a member of the research team. The discussion prompted the children to explain the choices and rankings they made in the classification activity to gain valuable insight into the rationale behind why and how they responded to the packages. The moderator also probed children for examples of aspects of packaging that they found important in making the cereal boxes or product packages more generally, appealing to them and other children. The same moderator conducted all focus group discussions following a semi-structured interview guide to ensure consistency in the interview and probing style between study sessions. Audio recordings and handwritten notes were taken during the discussions and were subsequently transcribed and imported into the NVivo 12 Plus© (version 12.6.0.959) software for analysis.

### 2.6. Analyses

#### 2.6.1. Quantitative Analyses

The percent agreement and Cohen’s Kappa statistic (κ) tested the pairwise agreement between the CAP tool’s and the children’s binary classifications of products with and without child-appealing packaging (i.e., binary outcome). Values of κ were interpreted as follows: Values ≤0 indicating “no agreement”, 0.01–0.20 as “none to slight”, 0.21–0.40 as “fair”, 0.41–0.60 as “moderate”, 0.61–0.80 as “substantial”, and 0.81–1.00 indicating “almost perfect agreement” [40]. A higher percent/pairwise agreement suggests higher criterion validity for this outcome. 

Spearman’s rank correlation tested the relationship between the CAP tool’s ranking of cereals based on marketing power scores and the children’s ranking of cereals based on their appeal (i.e., ranked outcome). Values of Spearman’s rho (*r_s_*) closer to +1 indicate a stronger positive relationship between the rankings, and therefore, higher criterion validity for this outcome. Values of *r_s_* were interpreted as follows: 0.00 as “zero”, 0.01–0.30 as “weak”, 0.31–0.60 as “moderate”, 0.61–0.99 as “strong”, and 1.00 as “perfect” [41]. 

The percent agreement, pairwise agreement, and correlation were analyzed overall, and in relevant subgroups (i.e., age group, gender, SES group). For the purposes of these exploratory subgroup analyses, children aged 5–8 years old were considered “younger” and children aged 9–13 years old were considered “older”. Gender was as declared by the participant. The SES group was defined based on the percentage of the population below the low-income measure after tax (% LIMAT) in the participant’s neighborhood of residence [29], determined by their postal code. Neighborhoods with ≤18.2% LIMAT were categorized as “lower” SES, 18.3–26.3% LIMAT were categorized as “middle”, and ≥26.4% LIMAT were categorized as “higher”. The sample size was underpowered to conduct these subgroup analyses as primary outcomes, but they were carried out for exploratory purposes to inform a future larger study. 

Cross-classification analyses were conducted between the CAP tool and children’s rankings (i.e., 1 to 6) of breakfast cereals according to the marketing power (CAP tool) and their appeal (children). Cross-classification analyses were conducted overall, and per the individual cereal box. The exact agreement was defined as the same ranking by both the CAP tool and children (e.g., the CAP tool scored the cereal box with the 2nd highest marketing power and children ranked it as their 2nd favorite). Agreement ±1 ranking (e.g., the CAP tool scored the cereal box with the 2nd highest marketing power and children ranked it as either their 1st or 3rd favorite) and disagreement (i.e., rankings ±2) were also calculated. If rankings were further apart than ±2 ranks, it was considered to be a gross misclassification. 

Quantitative analyses were completed in the RStudio© (version 1.1.463) and Microsoft^®^ Excel^®^ software.

#### 2.6.2. Qualitative Analyses

The quantitative outcomes related to criterion validity were supplemented with the qualitative evaluation of content validity through the analyses of the focus group discussions [25]. A conceptual content analysis (i.e., assessing the occurrence of concepts or terms in the data) [42,43] was conducted to assess the qualitative agreement between the marketing techniques included in the CAP tool and the marketing techniques and/or aspects of packaging that children highlighted as pertinent to them during the discussions.

Since our primary concern was how well the CAP tool techniques aligned with the aspects of packaging highlighted by children, a flexible deductive approach was taken for the content analysis, whereby codes were pre-determined based on the CAP tool (i.e., codes corresponding to each CAP tool technique), but additional codes could be incorporated to reflect new concepts, should they emerge [44,45,46]. Coding of transcripts was an iterative process, with one researcher coding and re-coding data until consistency and completion were achieved. 

The coding frequency of each code was calculated to determine the most common concepts (i.e., marketing techniques) in the data. The coded content was analyzed thematically to further interpret key concepts and ideas related to how children determine the food packaging’s child appeal. 

## 3. Results

### 3.1. Child-Appealing Packaging (CAP) Coding Tool

The final CAP tool includes 18 core techniques (Table A2) and 11 broad techniques (Table A3), for a total of 29 marketing techniques. Marketing power scores for products evaluated with the CAP tool could therefore range from 0 to 29, according to the number of marketing techniques that are displayed on the package. The full CAP coding tool is detailed in Appendix A.

### 3.2. CAP Tool Validation Study

All children completed binary classifications and ordered rankings for all six cereal boxes, resulting in 90 CAP-child binary cereal classification pairings and 90 cereal ranking pairings for analysis. 

#### 3.2.1. Criterion Validity (i.e., Quantitative Results)

Overall, there was an 80% agreement (n = 72/90 CAP-child pairings) between the CAP tool’s and children’s binary classification or the cereal boxes (e.g., with or without child-appealing packaging), resulting in “moderate” pairwise agreement (κ [95% CI]: 0.54 [0.35, 0.73]) (Table 3). Exploratory subgroup analyses found that in younger children there was a 76.4% agreement (n = 32/42 CAP-child pairings) resulting in “moderate” pairwise agreement (κ [95% CI]: 0.44 [0.14, 0.75]), while in older children there was an 83.3% agreement (n = 40/48 pairings), resulting in “substantial” pairwise agreement (κ [95% CI]: 0.63 [0.39, 0.86]). The “lower” SES status group had a 50% agreement (n = 9/18 CAP-child pairings) resulting in “no pairwise agreement” (κ [95% CI]: −0.08 [−0.58, 0.42]), the “middle” SES status group had an 83.3% agreement (n = 35/42 pairings) resulting in “substantial” pairwise agreement (κ [95% CI]: 0.62 [0.36, 0.88]), and the “higher” SES status group had a 93.3% agreement (n = 28/30 pairings) resulting in “almost perfect” pairwise agreement (κ [95% CI]: 0.84 [0.63, 1.05]). Analyses by gender were not possible due to the small and uneven sample sizes.

**Figure 1:**
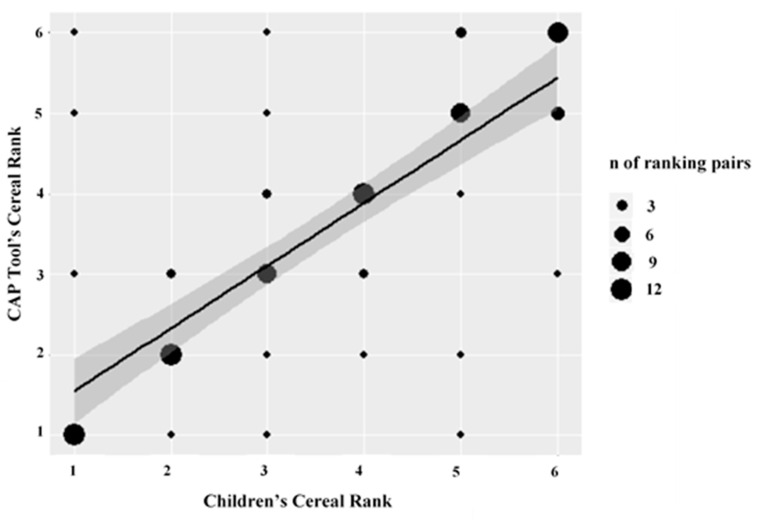
Spearman correlation (*r_s_*) between children’s ranking of cereal boxes in order of preference and the CAP tool’s ranking of cereal boxes according to the marketing power. The plotted line depicts the Spearman rank correlation and 95% CI between children’s ranking of cereals (i.e., 1 to 6) in order of preference and the CAP tool’s ranking of cereals (i.e., 1 to 6) in order of marketing power. Individual data points indicate the CAP-child ranking pairs (e.g., child cereal rank of “2”/CAP tool rank of “1”), with larger data points corresponding to a larger number of ranking pairs at that intersection.

There was a strong correlation (*r_s_* [95% CI]: 0.78 [0.63, 0.89], *p* < 0.001) between the CAP tool’s and children’s ranking of the cereal boxes (i.e., 1 to 6 in order of marketing power/appeal) (Figure 1). The median correlation across children was 0.94 and 46.7% (n = 7/15) of children ranked cereal boxes in perfect correlation (i.e., *r_s_* = 1) with the CAP tool’s evaluation of marketing power (data not shown). Exploratory subgroup analyses found that younger and older age groups showed a similar correlation between CAP and children’s cereal rankings (*r_s_* [95% CI]: 0.78 [0.51, 0.96], *p* < 0.001 and 0.78 [0.56, 0.92], *p* < 0.001, respectively) (Table 4). The “lower” SES status group had a correlation of 0.62 [0.19, 0.94], *p* = 0.006, the “middle” SES status group had a correlation of 0.79 [0.53, 0.97], *p* < 0.001, and the “higher” SES status group had a correlation of 0.86 [0.7, 0.96], *p* < 0.001. The correlation in all of the subgroups was strong. 

**Figure 2:**
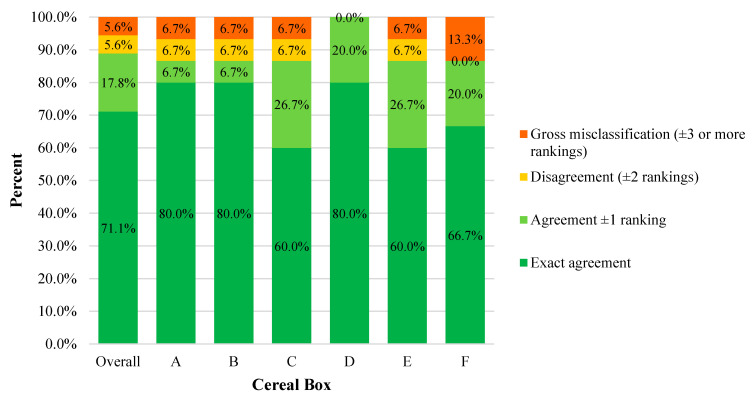
Cross-classification analyses of the agreement between children’s and the CAP tool’s ranking of cereal boxes, overall, and per cereal box. Cross-classification analyses were conducted between the CAP tool and children’s rankings (i.e., 1 to 6) of breakfast cereals according to the marketing power (CAP tool) and their appeal (children). Cross-classification analyses were conducted overall and per individual cereal box. Exact agreement was defined as the same ranking by both the CAP tool and children (e.g., CAP tool scored the cereal box with the 2nd highest marketing power and children ranked as their 2nd favorite). Agreement ±1 ranking (e.g., CAP tool scored the cereal box with the 2nd highest marketing power and children ranked as either their 1st or 3rd favorite) and disagreement (i.e., rankings ±2) were also calculated. If rankings were further apart than ±2 ranks, it was considered to be gross misclassification. Cereal boxes are ranked in order of least (A) to most (F) powerful marketing.

Cross-classification analyses resulted in exact agreement for 71.1% (n = 64) of CAP-child cereal ranking pairings, 17.8% (n = 16) had agreement ±1 ranking, and there was disagreement and gross misclassification for 5.6% (n = 5) of pairings, each (Figure 2). The exact agreement ranged from 60–80% across individual cereals, agreement ±1 ranged from 6.7–26.7%, disagreement ranged from 0–6.7%, and gross misclassification ranged from 0–13.3%.

#### 3.2.2. Content Validity (i.e., Qualitative Results)

**Figure 3:**
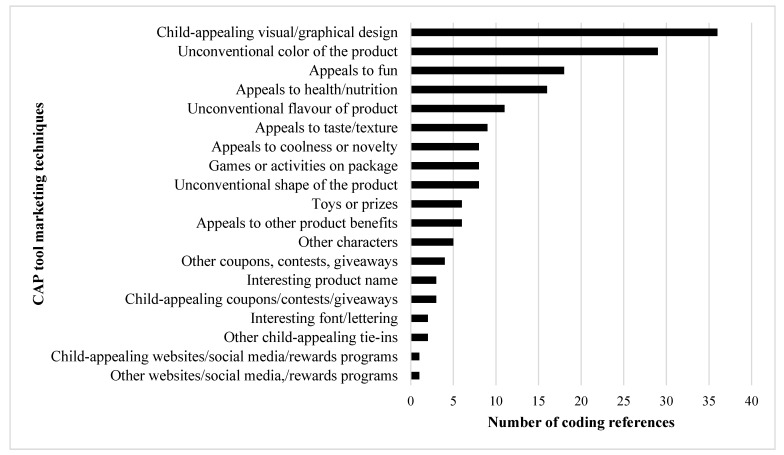
Number of coding references for each CAP tool marketing technique discussed by children during focus groups. Marketing techniques from the CAP tool were used as codes to analyze the transcripts from the focus group discussions with children. The number of times each marketing technique was mentioned by children was counted. A full description of the marketing techniques can be found in Appendix A.

The content analysis of the focus group transcripts showed that 19/28 CAP marketing techniques were discussed. This included 12/18 core techniques and 7/10 broad techniques. The techniques most frequently mentioned by children were child-appealing visual and graphical design (36 times), unconventional color of the product (29 times), and appeals to fun (18 times). It is important to note as well that children did not discuss any techniques or aspects of packaging that were not covered by the CAP tool. The frequency of all coded techniques is shown in Figure 3. Overall, qualitative analyses suggest that the CAP tool has content validity in that it measures child-appeal using a list of techniques that correspond to the aspects of packaging that children discussed in the focus groups. 

Three key themes emerged from the content analysis related to the aspects of packaging that drove its appeal to children. Firstly, it was clear that the children were drawn to cereal boxes (and food packaging more generally), that were, in their words: “Fun”, “cool”, “exciting”, and “interesting”. They thought that packages that displayed these characteristics (e.g., “fun”, “cool”) were meant for them or other kids. Conversely, children thought that if cereals were “boring and plain”, “not really exciting” or if “they just look like normal cereal”, they were not meant for kids and should be for adults. These notions served as explanations for many of the children’s decisions on whether a particular cereal box was “for kids” or not. Children were adamant that aspects of the package’s design, such as color, the presence of characters, and a “kiddie atmosphere” were key for building its overall appeal (or how “fun” it was). Moreover, participants discussed that if the cereal “[had] games on it” or “free toys”, this made it seem fun and therefore appealing. Having a cereal that “looks yummy” was also key in driving a cereal’s attractiveness, according to children. Many of the aspects of packaging that children mentioned most often as generating appeal or making them think a cereal was “for kids” correspond to the core marketing techniques in the CAP tool, examples of which are shown in Table 5. Importantly, it was apparent from the discussions that cereal boxes which were lacking these core techniques and were more “plain” or “dull”, were not nearly as interesting or attractive to children.

The second theme that emerged from the discussions was that children were drawn to more than just the typical child-appealing marketing techniques on the cereal boxes. Many of the broad techniques included in the CAP tool were also raised unprompted by participants as aspects of packaging that are important or attractive to them. For example, children mentioned various appeals to health and nutrition, however, whether this made them think a product was “for kids” was contentious. Some participants interpreted nutrition claims and other nutrition marketing as “fun facts” to have on children’s food, while others perceived “looking healthy” as something meant for adults, and therefore not as important to them. Regardless, children were clearly drawn to aspects of health on product packaging and paid close attention to these marketing techniques. Children were also drawn to the concept of value, a characteristic they felt increased the attractiveness of the product. Children discussed value based on several different aspects, such as price, the size of the product or “if you feel like you get something for buying it”, such as a prize. Participants also noted broad marketing techniques such as social media logos and giveaways that were not necessarily intended for children, discussing a prepaid gas card, in this instance as something of value to both them and their parents. Table 6 illustrates the pertinence of these broad marketing techniques.

Lastly, the discussions highlighted that the more “different things on [the box]” increased the “interestingness” of the cereal, and therefore its child-appeal. Contrarily, participants discussed that on plain or “dull” packages, “there’s no detail so its just less interesting” to them. Put simply, the more “fun” and the less “boring” a package was, the more appealing it was to children. Participants explained that cereal boxes that looked more cool, more interesting, more exciting or a package that “pops out on the shelf and doesn’t fall back in all the brands”, are the ones they liked best. As discussed, there were several aspects of packaging that made a cereal stand out to kids, and consequently made them rank it higher than others during the activity. It was clear from the discussions that displaying multiple core techniques on product packaging was critical to creating a strong child appeal, and that lacking these core techniques made products less attractive to participants. Moreover, it became apparent that broad marketing techniques, while perhaps not as immediately salient and displayed alone, when appearing in combination with core techniques, were responsible for generating additional interest from children and ultimately building the package’s overall appeal. 

## 4. Discussion

This study resulted in the development of the CAP coding tool, a novel methodology for measuring the presence, type, and power of child-appealing marketing on packaged food and beverage product packaging, based on a published inventory of marketing techniques. Therefore, the CAP tool evaluates marketing techniques on food packages using a comprehensive, evidence-based set of core and broad marketing techniques to capture the full breadth of “child-appeal” across three primary outcomes, the validity of which were tested in this pilot study. 

The goal of this pilot mixed-methods validation study was to evaluate the criterion and content validity of the CAP tool based on how children perceive marketing on product packaging. The results suggest that participants primarily thought cereals were “for kids” when they displayed core marketing techniques, and when they did not, participants did not feel these cereals were intended for them. The 80% agreement and “moderate” pairwise agreement between children’s and the CAP tool’s categorization of cereals as “child-appealing” was corroborated by the qualitative results which found that the display of core techniques was critical in driving the attractiveness of packaging for children. These findings suggest the validity of the CAP tool’s binary measure of child-appealing packaging based on the display of one or more core techniques. This study also found validity in the CAP tool’s marketing power score, defined by the number of marketing techniques displayed on the package. Quantitative results found a “strong” correlation between children’s ranking of cereal boxes in order of preference and the CAP tool’s ranking of cereal boxes according to the marketing power, with almost half of participants ranking in perfect correlation with the CAP tool. Moreover, each individual cereal was ranked either in perfect agreement between children and the CAP tool or within ±1 ranking, 88.9% of the time. The analysis of the focus group discussions confirmed that children were in fact more interested in packages that displayed more marketing techniques, and vice versa. While there were a very small number of gross-misclassifications (i.e., ≥3 rankings) in cereal rankings, these were not corroborated during the focus group analyses and results therefore suggest that the CAP tool’s marketing power score is in line with children’s attraction to packages with varying degrees of marketing. Additionally, two-thirds of the CAP tool marketing techniques were discussed by children at least once during this pilot study, suggesting that the types of marketing techniques included in the coding tool are in line with those that children find salient. It is worth acknowledging that one third of core techniques (n = 6/18) were not mentioned by children, inferring that those techniques are perhaps not actually critical in driving child-appeal. However, the CAP techniques that were not mentioned (i.e., presence of branded characters, licensed characters, celebrities, children/parents/families, and recipes (specifically appealing to children)) were not featured on the mock cereal packages and so may not have been specifically triggered in the minds of the children in this study. However, these unmentioned techniques are similar to techniques that were mentioned in the focus groups (e.g., “other characters”) and are techniques that are frequently measured in the literature and found on food packages [16,17,47]. Therefore, these techniques were retained as core techniques in the CAP tool, but warrant further investigation in future studies. Importantly, qualitative analyses found that children were drawn to aspects of packaging other than typical child-appealing marketing techniques, namely, the CAP tool’s broad techniques. These findings justify the inclusion of broad techniques within the CAP tool and its marketing power score, as these techniques, when displayed in combination with core techniques, are integral to building the overall appeal of the package—seen in children’s consistently higher ranking of cereals displaying stronger marketing. This speaks to the importance of evaluating both core and broad marketing techniques as part of future child-appealing marketing monitoring activities, given that both seem to garner the attention of children. 

The results of this work align with previous studies analyzing children’s perceptions of food packaging. Previous research has highlighted the importance of core marketing techniques in creating product packaging that is appealing to children, such as the use of characters; color, and other visual elements; premiums and giveaways; games and activities; celebrity or athlete endorsements; etc. [16,17,18,47,48,49]. The display of such techniques has also been shown to alter children’s perception of taste and increase their attraction to the product [48,50,51,52], and have been further shown to translate into purchase requests and consumption patters [50,51,52,53,54,55,56]. This body of evidence in the literature corroborates this study’s findings that core techniques are critical to building children’s interest in products and in determining which food products or instances of marketing should be considered “child-appealing”. Additional studies have examined children’s perceptions of broad marketing techniques, in particular, the use of appeals to health or nutrition. Nutrition marketing and nutritional components have been found to increase children’s preference and/or choice of foods [48,56,57,58,59]. Others have found that packaging that “looks healthy” has a deterrent effect [60]. Regardless, these studies have shown that these aspects of packaging do not go unnoticed by children, despite not being specifically intended to appeal to them. These findings align with the results of the current study that found that appeals to nutrition and health, as well as other broad, non-children-specific marketing techniques were salient to children when observing packaged foods. This is an important consideration for policymakers if they wish to develop restrictions that will encompass the full spectrum of child-appealing marketing. Overall, this study builds on the broader body of literature by applying understandings of marketing on food packaging in a novel manner, by validating a coding tool to quantitatively measure child-appeal, in line with what children actually find appealing. Despite the preliminary nature of these results, their alignment with previous literature further supports the validity of the CAP tool in measuring child-appeal and marketing power. 

There were several strengths to this validation study, particularly its use of a mixed methods approach, which allowed for a more comprehensive analysis whereby the qualitative data supplemented and provided additional context to the quantitative analysis. Moreover, the use of professionally designed, non-branded, nutritionally uniform mock cereals reduced several sources of bias in children’s classification and ranking of the packages. Limitations arose due to the pilot nature of this study, particularly the small sample size and uneven gender ratio, and as such, the results of this work should be interpreted with caution. Some evidence has noted the varied influence of child-appealing marketing across different demographics of children [17,61,62,63,64,65] and while these exploratory results suggest potential differences across age and SES groups in terms of the CAP tool’s validity, this study was not adequately powered for subgroup analyses. The primary study plans to have an expanded sample size to allow for analyses to elucidate potential age, SES, and/or gender-based differences in what constitutes “child-appeal”. Moreover, while this study had a relatively diverse sample in terms of age and SES, the thoughts and opinions reflected here are limited to those of the children in this urban Toronto cohort and may not be generalizable to the views of children more broadly in Canada or internationally. Additionally, while children were probed for their opinions on food packaging more generally, this study only tested the validity of the CAP tool using marketing on one type of food product (i.e., cereals) and it is possible that the concept of “child-appeal” could vary in different food groups (e.g., candy or junk foods, which could be inherently appealing to children, regardless of packaging). Lastly, while qualitative coding and analysis was conducted multiple times to ensure consistency and accuracy, it was carried out by a single researcher and may have incurred bias as such. The primary study will use dual coding by two independent researchers.

The novel coding tool that was validated in this study is further strengthened by its basis on a published inventory of marketing techniques used in child-appealing marketing research [16]. The techniques in the CAP tool also appear to mirror those covered in existing or emerging children’s marketing regulations, with the addition of broad techniques that are important to monitor due to their contribution to marketing power. It is important to note, however, that that the CAP tool’s marketing power score weighs the presence of each technique equally (i.e., one point each), but it is possible that certain marketing techniques are more persuasive to children than others [17,21], and therefore contribute disproportionately to the power and influence of the marketing message. The power of specific marketing techniques in relation to—or in combination with—other techniques was not tested here and should be the aim of future research. The CAP tool could be easily adjusted to reflect the emergence of new evidence, and this methodology is nonetheless an important step in quantifying the persuasiveness of child-appealing marketing. Additionally, while this study focused on criterion and content validity, future studies should aim to test other types of validity (e.g., face validity with experts) to further validate this tool. Moreover, while the concepts that have been validated in this study could be extended to other marketing mediums, future research should aim to validate coding methodologies specific to other mediums to account for differential effects of marketing across marketing platforms. However, the specific marketing techniques in the CAP tool could be adapted to reflect the marketing techniques used on different marketing platforms (e.g., television, websites, etc.) or evolving marketing practices more generally, and could therefore have applicability in many current and future research settings.

## 5. Conclusions

Overall, the results of this mixed-methods pilot study suggest that the CAP coding tool may be a valid methodology for measuring the presence, type, and power of child-appealing marketing on product packaging, based on the core and broad marketing techniques that children actually find appealing. Further research on a larger, more diverse sample of children and examinations of additional types of validity are needed for full validation. Regardless, the CAP tool presents an important methodological improvement in this field and could prove useful in future research, policy development, and child-appealing marketing monitoring activities.

## Figures and Tables

**Table 1:** Outcome variables of the child-appealing packaging (CAP) coding tool.

Outcome Variable	Explanation	Details and Derivation
Presence of child-appealing marketing	Determines whether the product packaging is child-appealing, based on the display of core marketing techniques.	Binary Variable (i.e., Yes (child-appealing packaging): ≥1 core marketing technique displayed; No (not child-appealing): 0 core techniques displayed).
Type of child-appealing marketing	Determines which specific type(s) of core or broad marketing technique(s) is being displayed.	Presence (binary) or frequency (count) of individual core or broad marketing techniques displayed within a sample.
Power of child-appealing marketing	Determines the power (persuasiveness) of the marketing message based on the number of unique core and broad marketing techniques displayed.	Marketing power score (count variable): Sum of all the unique core and broad techniques displayed on the package (e.g., 1, 2, 3, etc.).

**Table 2:** Demographic characteristics of participants (n = 15 children).

Gender	n (%)
Female	3 (20%)
Male	12 (80%)
Socioeconomic status (SES) group ^1^	n (%)
Lower	3 (20%)
Middle	7 (47%)
Higher	5 (33%)
Age Group ^2^	n (%)
Younger	7 (47%)
Older	8 (53%)
Mean age	8.7 years
Age range	5–13 years

^1^ The SES group was defined based on the percentage of the population that was below the low-income measure after tax (%LIMAT) in the participant’s neighborhood of residence (according to the 2016 City of Toronto Census data: https://www.toronto.ca/city-government/data-research-maps/neighbourhoods-communities/neighbourhood-profiles/ (accessed on 10 February 2020)) determined by their postal code. Neighborhoods with ≤18.2% LIMAT were categorized as “lower” SES, 18.3–26.3% LIMAT were categorized as “middle”, and ≥26.4% LIMAT were categorized as “higher”. ^2^ Children aged 5–8 years old were considered “younger” and children aged 9–13 years old were considered “older”.

**Table 3:** Percent agreement and pairwise agreement between children’s and the CAP tool’s categorization of cereal boxes as “child-appealing” or not.

	Percent Agreement % (n Pairings) ^1^	Pairwise Agreement κ (95% CI) ^2^	κ Interpretation ^3^
Overall (n = 15)			
	80% (n = 72/90)	0.54 (0.35, 0.73)	“Moderate agreement”
Socioeconomic status (SES) group ^4^			
Lower (n = 3)	50% (n = 9/18)	−0.08 (−0.58, 0.42)	“No agreement”
Middle (n = 7)	83.3% (n = 35/42)	0.62 (0.36, 0.88)	“Substantial agreement”
Higher (n = 5)	93.3% (n = 28/30)	0.84 (0.63, 1.05)	“Almost perfect agreement”
Age group ^5^			
Younger (n = 7)	76.4% (n = 32/42)	0.44 (0.14, 0.75)	“Moderate agreement”
Older (n = 8)	83.3% (n = 40/48)	0.63 (0.39, 0.86)	“Substantial agreement”

^1^ Percentage of cereals categorized similarly by both children and the CAP tool, and the number of CAP-child pairings categorized the same way, overall, and in subgroups. ^2^ Pairwise agreement was tested using Cohen’s Kappa Statistic (κ). ^3^ Values of κ were interpreted as follows: Values ≤0 indicating “no agreement”, 0.01–0.20 as “none to slight”, 0.21–0.40 as “fair”, 0.41–0.60 as “moderate”, 0.61–0.80 as “substantial”, and 0.81–1.00 indicating “almost perfect agreement”. ^4^ The SES group was defined based on the percentage of the population that was below the low-income measure after tax (%LIMAT) in the participant’s neighborhood of residence (according to the 2016 City of Toronto Census data: https://www.toronto.ca/city-government/data-research-maps/neighbourhoods-communities/neighbourhood-profiles/ (accessed on 3 February 2020)) determined by their postal code. Neighborhoods with ≤18.2% LIMAT were categorized as “lower” SES, 18.3–26.3% LIMAT were categorized as “middle”, and ≥26.4% LIMAT were categorized as “higher”. ^5^ Children aged 5–8 years old were considered “younger” and children aged 9–13 years old were considered “older”.

**Table 4:** Spearman’s rank correlation (*r_s_*) between children’s ranking of cereal boxes in order of preference and the CAP tool’s ranking of cereal boxes according to the marketing power, overall, and among socioeconomic status and age subgroups.

	*r_s_* (95% CI) ^1^	*p*-Value ^2^	*r_s_* Interpretation ^3^
Overall (n = 15)			
	0.78 (0.63, 0.89)	<0.001	“Strong correlation”
Socioeconomic status (SES) group ^4^			
Lower (n = 3)	0.62 (0.19, 0.94)	0.006	“Strong correlation”
Middle (n = 7)	0.79 (0.53, 0.97)	<0.001	“Strong correlation”
Higher (n = 5)	0.86 (0.70, 0.96)	<0.001	“Strong correlation”
Age group ^5^			
Younger (n = 7)	0.78 (0.51, 0.96)	<0.001	“Strong correlation”
Older (n = 8)	0.78, (0.56, 0.92)	<0.001	“Strong correlation”

^1^ Spearman’s rank correlation (*r_s_*) and 95% CI between children’s ranking of cereals (i.e., 1 to 6) in order of preference and the CAP tool’s ranking of cereals (i.e., 1 to 6) in order of marketing power. ^2^ *p*-values <0.05 were considered to indicate an *r_s_* significantly different than zero. ^3^ Values of *r_s_* were interpreted as follows: 0.0 as “zero”, 0.01–0.3 as “weak”, 0.31–0.6 as “moderate”, 0.61–0.99 as “strong”, and 1.0 as “perfect”. It has been suggested that a correlation coefficient >0.7 can be considered “strong”. ^4^ The SES group was defined based on the percentage of the population that was below the low-income measure after tax (%LIMAT) in the participant’s neighborhood of residence (according to the 2016 City of Toronto Census data: https://www.toronto.ca/city-government/data-research-maps/neighbourhoods-communities/neighbourhood-profiles/ (accessed on 3 February 2020)) determined by their postal code. Neighborhoods with ≤18.2% LIMAT were categorized as “lower” SES, 18.3–26.3% LIMAT were categorized as “middle”, and ≥26.4% LIMAT were categorized as “higher”. ^5^ Children aged 5–8 years old were considered “younger” and children aged 9–13 years old were considered “older”.

**Table 5:** Examples of quotes illustrating children’s discussion of core marketing techniques ^1^.

**Examples of Quotes Discussing Core Marketing Techniques, Explaining Why Children Liked Some Cereal Boxes More Than Others:**
“It looked like they have little sugar-coated colorful thingies that kids love, you know, like those fruit loops and stuff that have all those colorful rings”
“And there’s like a zombie kind of...and it says like ‘Sooo much fun!’”
“Because there’s a lot of colors”
“I like the tic tac toe”
“Usually a lot of people’s eyes go to the more colorful things and there’s the one that said uhm, ‘free toy inside’ which would be more kids oriented and ‘cause adults don’t really like toys”
“Uh because it looks cool...so kids would probably get tricked into eating it cause its like ‘Yeah, I wanna be a cool kid I’m gunna pick this cereal!’”
“If it has like lots of pictures or like the colors of the pictures or the colors of the food and its not just like regular food or just plain”
“[Kids like cereals that] make them have fun because they’re so colorful and it makes them excited”
“Its quite interesting to see they’re both from the same brand and one has more like kiddie atmosphere and all these colors and this one is like for parents and adults and older people and stuff [*indicating less powerfully marketed cereals*]”
“Yeah, it says like you get a free toy and “cool new colours” like that...and uhm on this one [*indicating a more powerfully marketed box*] it there it says: “guaranteed great taste” but this one [*indicating a less powerfully marketed box*] says nothing in large and there’s no detail so its just less interesting… and look it says: “kids club” there!”
“Interestingness! [*When probed to explain what they meant by “interestingness”:*] “Like lots of different things on it, like games.”
“I’m looking at what pops out on the shelf, like what doesn’t fall back in all the brands” [*When probed on what makes something ‘pop out’:*] “More color!” “Uhm interesting names” “Free toys!” “Any catchy names of the brands”
**Examples of quotes discussing the lack of core marketing techniques, explaining why children disliked some cereal boxes more than others:**
“If its boring and plain…usually like, kids like something that’s more like hilarious n stuff”
“They were just normal cereals with fruits in them”
“Cause there’s not as much color as the kids ones cause they usually put A LOT of color”
“This one doesn’t have a like kids picture [*referring to cartoon characters*] or looks like more for adults…They look boring”

^1^ Text written in italics represents additional explanation provided by the researchers to assist in the interpretation of the quotes.

**Table 6:** Examples of quotes illustrating children’s discussion of broad marketing techniques.

**Examples of Quotes Discussing Broad Marketing Techniques, Explaining Why Children Liked Some Cereal Boxes More Than Others:**
“Oh the prepaid 5$ gas card, the parents are gunna want their kids to get that so that they can get free stuff”
“They had a lot of like uhm like facts like “high cholesterol is a risk” but the adult ones just had nothing on it” [*When probed on if they think nutrition marketing is interesting:*] “Yeah, like fun facts.”
“Maybe buy one get one free?”
“The size. Like how big it is or how small it is” [*When probed on what size packages children prefer:*] “Bigger” “Big!” “This one is small so adults would like it better…this one’s even smaller!”
“Yeah, cause over there [*pointing at a plain box*] it doesn’t say “value” or anything” [*When probed on whether ‘good value’ makes them like the cereal more:*] “Yeah definitely.”
“When you feel like you get something back like a 5$ prepaid gas card and if you feel like you get something for buying it”
**Examples of quotes discussing broad marketing techniques, explaining why children disliked some cereal boxes more than others:**
“If they’re expensive and lame”
“It can’t just say like 5000 calories” “That would be a lot”
“Some kids like are smart they look over here [pointing at Nutrition Facts Table]” [*When probed on what type of ‘nutrition’ children look for:*] “Unhealthy!” “Super unhealthy!”
“If one costs a hundred dollars!”
“Looks like something you would take for a diet or something”

Text written in italics represents additional explanation provided by the researchers to assist in the interpretation of the quotes.

## Data Availability

The data presented in this study are available on request from the corresponding author.

## Supplementary Materials

The following are available online at https://www.mdpi.com/article/10.3390/ijerph18094769/s1. Supplementary Table S1: Mock cereal boxes used in child-appealing packaging (CAP) coding tool validation study.

## Appendix A. Child-Appealing Packaging (CAP) Coding Tool

**Development and purpose**

The child-appealing packaging (CAP) coding tool was developed as a novel methodology to measure the presence, type, and power of child-appealing marketing on product packaging, by evaluating the marketing techniques displayed on the package. The CAP tool was developed based on a published inventory of marketing techniques that have previously been used in child-appealing marketing research [16]. The CAP tool includes marketing techniques that are popular on product packaging specifically (e.g., cartoon characters, toys in the box), as well as techniques that have traditionally been used in other marketing platforms but are now appearing on packaging with evolving marketing practices (e.g., social media handles, scannable codes linked to websites). Marketing techniques included in the CAP tool were categorized into two categories: core techniques and broad techniques, described in detail further below.

**CAP coding tool outcome variables**

There are three primary outcome variables of the application of the CAP tool related to the presence, type, and power of child-appealing marketing, detailed in Table A1. Briefly, the CAP tool measures the presence of child-appealing marketing (i.e., if the package is child-appealing or not), based on the display of one or more core marketing technique(s), and captures the type of the marketing based on the presence or frequency of display of individual core/broad marketing techniques within the sample. Finally, the CAP tool scores marketing power by summing all the techniques (core and broad) displayed on the package, based on evidence that the number of marketing techniques displayed on the package increases the persuasiveness or intensity of the marketing message [19,20].

**Table A1:** Outcome variables of the child-appealing packaging coding tool.

Outcome Variable	Explanation	Details and Derivation
**Presence of child-appealing marketing**	Determines whether the product packaging is child-appealing, based on the display of core marketing techniques.	Binary Variable (i.e., Yes (child-appealing packaging): ≥1 core marketing technique displayed; No (not child-appealing): 0 core techniques displayed)
**Type of child-appealing marketing**	Determines which specific type(s) of core or broad marketing technique(s) are being displayed.	Presence (binary) or frequency (count) of individual core or broad marketing techniques displayed within a sample
**Power of child-appealing marketing**	Determines the power (persuasiveness) of the marketing message based on the number of unique core and broad marketing techniques displayed.	Marketing power score (count variable): sum of all unique core and broad techniques displayed on the package (e.g., 1, 2, 3, … etc.)

**Core marketing techniques**

Core techniques are marketing techniques that could independently make a package appealing to children (e.g., cartoon characters or games on the package). Core techniques are also those that are typically included in marketing regulations or restrictions, as these are more ‘objectively’ or defensibly appealing to children.

If a product’s packaging displays one or more of these techniques, then it will be considered to have “child-appealing packaging”. It is important to ensure that that ALL techniques that are displayed are coded (i.e., 1 = present/0 = absent), as the use of multiple techniques increases marketing power score.

**Table A2:** Core marketing techniques, definitions, and examples.

#	Technique	Definition	Examples
**1**	Child-appealing visual/graphical design of package	Intense colors, patterns or visual designs on the packaging or design themes related to fantasy, adventure, magic, sports, etc. that are clearly appealing to children. *Note: this can include child-appealing lettering, if it is enough on its own for the product to be considered “child-appealing”, otherwise code lettering under broad techniques.*	Space-themed visual design Rainbow packaging Chalkboard-style lettering
**2**	Unconventional shape of the product, featured on the package	The product featured on the packaging has a shape that is unconventional or unusual for that type of product. E.g. if crackers have a shape other than their usual square or round shape. *Note: In the case of clear plastic containers where the product is visible through the package, this counts as the shape being visible.*	Animal shaped crackers Alphabet shaped pasta Character, fruit or animal shaped gummies
**3**	Unconventional flavour of the product, featured on the package	The product featured on the package has a flavour that is unconventional or unusual for that type of product, or a flavour that is not a ‘real’ or ‘discernable’ flavour. *Note: this could include the presentation of the flavour in a ‘negative’ way that may appeal to children; e.g., tastes crazy, weird, sour, whacky*	Tropical Storm Flavour Cheddarific Secret Flavour Chocolate Mud flavour Cool Cucumber flavour
**4**	Unconventional colour of the product, featured on the package	The product featured on the package has a colour that is unconventional or unusual for that type of product. E.g. if crackers are coloured rather than their usual plain/brown colour. *Note: In the case of clear plastic containers where the product is visible through the package, this counts as the color being visible.*	Rainbow crackers Purple Ketchup Colour changing drink powder Rainbow fruit roll ups (instead of just red, for example) *Note: multi-colored candies would NOT be unusual, unless they are described in a more ‘fun’ or child-appealing way.*
**5**	Games or activities on package	Presence of games or activities on the package.	Connect the dots Mazes “Count how many snowmen”
**6**	Presence of branded characters or spokespersons	Presence of company- or brand-owned characters.	Tony the Tiger Toucan Sam Cap’n Crunch Kraft Bears Pillsbury Doughboy
**7**	Presence of Licensed Characters	Presence of characters from TV shows, movies, books, etc., that may appeal to children. Note: human actors, if presented as the character are included here (e.g., Miley Cyrus as Hannah Montana), if portrayed as themselves, include under “Presence of Celebrities” (e.g., Miley Cyrus advertised as Miley Cyrus).	Dora the Explorer Batman Hannah Montana Star Wars characters
**8**	Presence of celebrities	Presence of actors, athletes, musicians, other public figures that may appeal to children	Derek Jeter Miley Cyrus
**9**	Other characters or cartoons	Presence of cartoon characters, animals, etc. that are not branded, licensed, celebrities or tie-ins to child-appealing media (i.e., that do not fit into any of the above techniques)	Cartoon pictures of fictional sports players Animal cartoons on animal crackers
**10**	Other child-appealing tie-ins	Other movie/sports/TV show etc. tie-ins that are appealing to children are advertised on the package aside from one of the types of characters or celebrities described above. *Note: these may appear in addition to the presence of any characters described above*	Hockey tie-ins that feature an ice-rink or hockey equipment with/without a specific player. Harry Potter tie-in where Hogwarts is presented with/without a character.
**11**	Presence of children/parents/families	Presence of children or children with their families on the package, either real people or cartoon.	Children shown eating the product Pictures of children eating with their parents
**12**	Toys or prizes	Toys or prizes included with or inside the package or to be redeemed later.	Figurine inside package Stickers inside package
**13**	Coupons, contests, or giveaways, specifically appealing to children	Coupons, contests or giveaways to be entered or redeemed later. *Note: contests or giveaways must be for child-appealing prizes (unlike, for e.g., a Patio Furniture set)*	Enter to win tickets to a child-appealing movie Coupon for free yogurt tubes
**14**	Children’s product lines, featured on the package	A product line that is designed/branded for children is featured/named on the package, either for that product, or a different product.	“mini-” or “junior” product lines (e.g., Minigo yogurt) Lunchables “Small cookies for small hands”
**15**	Appeals to fun	Product packaging makes appeals to the product being fun or funny, having fun while eating the product, being happy, enjoyment, humour etc. *Note: this includes “fun” packaging (i.e., Packaging that is designed in a way to promote “fun” during eating, or makes eating an “activity”)* *Note: this could be as part of the product name (e.g., “Fun Dip”), if it is clearly “fun” and appealing to children*	“Have more fun with” “Feel the bubbles melt” “Try our crazy new flavors” “Smiles included” Display of children having fun, being happy, enjoying the product Yogurt Tubes Dunkaroos Processed cheese with dipping breadsticks (if “dipping” is promoted as an activity)
**16**	Appeals to coolness or novelty	Product packaging makes appeals to the product being cool/hip or new, being cool, while eating the product, etc. *Note: this could be as part of the product name; e.g., “Kool Kreatures”*	“Try our crazy new flavors” Kool-Aid “Try me!” On-pack claim that the product is “new”
**17**	Recipes, specifically appealing to children	Product packaging displays recipes that can be made using the product and may appeal to children or are promoted as appropriate for children or families to make together.	Rice Krispy squares Party Snack Mix
**18**	Promotion of websites, social media, rewards programs, specifically appealing to children	Product packaging promotes product/brand/company website, child-specific or games-based brand website, social media, or opportunities to “join”, “become a member”, redeem points, and collect rewards or to connect or share with others in a manner that is evidently child-appealing	“Find more games on [website]” References to “kids club” or similar

**Broad marketing techniques**

Broad techniques are marketing techniques that would not on their own cause a product to be considered as “child-appealing”; however, in addition to the core techniques, these could increase the power of the marketing message as a whole. Evidence has shown that marketing techniques such as promoting a product’s health, nutritional or economic value were amongst the most popular techniques used in child-appealing marketing research, despite them not being typical child-appealing techniques [16]. Broad techniques also include marketing techniques that may not appeal directly to children but may appeal to their parents or caregivers (e.g., product benefit claims, convenience packaging), and therefore be purchased for children. These techniques are important to monitor, given that when/if child-appealing marketing is restricted, a proliferation of broad techniques may occur as a means for manufacturers to circumvent regulations and ensure that their products are still consumed by children.

It is important to ensure that ALL techniques that are displayed on the package are coded (i.e., 1 = present/0 = absent), as the use of multiple techniques increases the marketing power score. Broad techniques should be coded even on products that will NOT ultimately be considered to have child-appealing packaging (i.e., displaying ≥ 1 core techniques) so that their use can be monitored over time.

**Table A3:** Broad marketing techniques, definitions, and examples.

#	Technique	Definition	Example
**19**	Interesting font or lettering	Presence of product name or description (e.g., product flavour) written or designed in a colorful, creative, or interesting font that is not on its own enough to make the package “child-appealing”, but may contribute to the overall power of the marketing *Note: this broad technique exists due to the difficult nature of identifying child-appealing lettering, and since often products will use bubbly or colorful fonts, but this alone is not always enough to consider a product child-directed.*	Aero bar bubble lettering Corn Pops lettering Cheetos lettering
**20**	Interesting or unconventional product name	Unconventional product name (e.g., strange spelling, rhyming, and alliteration) that may be interesting to children and build marketing power, *Note: if not counted as part of a core technique (e.g., appeals to fun or appeals to coolness/novelty) and not enough to make the product child-appealing on its own.*	Frooty Hoops Juicy Jels Wagon Wheels “Eat the middle first”
**21**	Presence of a logo not specifically appealing to children	Presence of a product/brand logo that is not specifically appealing to children. This includes when branded characters or spokespersons are used as part of the logo. *Note: presence of branded characters NOT within the product logo are included in the core technique: “presence of branded characters”*	The man with a moustache in the Pringles logo Quaker Oats man in the logo
**22**	Convenient packaging	Package is designed in a way to promote for easy or convenient packing, on-the-go snacking, or individually packaged servings. *Note: if the packaging is promoted as “fun” or as an activity, count under “appeals to fun”*	Processed cheese with dipping breadsticks Tuna and crackers kit Processed cheese with dipping breadsticks (if “dipping” is NOT promoted as an activity) Small yogurt containers “Great for packing in lunches”
**23**	Appeals to taste or texture	Product packaging makes appeals to the flavour taste, or texture, of the product, in a way that is not specifically appealing to children.	“New look, same great taste” “You’ll love it” “Delicious!” “Tastes like mama made it” Promotion of textures (e.g., crunchy, smooth…)
**24**	Appeals to health or nutrition	Product packaging makes appeals to the healthfulness or nutritional quality of the product, its ability to promote growth, strength, or physical activity. Product packaging displays “healthy foods” alongside the product. *Note: includes health and nutrition claims/symbols, as well as organic or natural claims/symbols*	“Helps them grow strong” “Part of a healthy breakfast” Fruit featured beside the product on pack (e.g. bowl of strawberries beside cereal) Source of 5 whole grains Made with 100%... Promotion of ‘real’, ‘pure’, ‘natural’ etc.
**25**	Appeals to other product benefits	Product packaging makes appeals to other product benefits aside from health/taste/fun. For example, value, quickness, easy preparation, sustainability, philanthropy, enjoyment while eating, etc. *Note: this does not include small statements (often on the bottom of the package) that the package was made from recycled materials or is recyclable.*	“Quick and easy” “Ready in 5 minutes” “Ready to bake” Proceeds go to X organization “Enjoy it, you deserve it!”
**26**	Recipes, not specifically appealing to children	Product packaging displays recipes that can be made using the product and do not specifically appeal to children/families	Bran muffins Low calorie smoothies
**27**	Promotion of websites, social media, rewards programs, not specifically appealing to children	Product packaging promotes product/brand/company website, social media, or opportunities to “join”, “become a member”, redeem points, and collect rewards or to connect or share with others, in a way that is not specifically child-appealing *Note: does not include link to company/manufacturer website included as part of contact information on package*	Social Media links Links to recipe websites Links to “create the next flavour of chips” QR codes
**28**	Coupons, contests, or giveaways, not specifically appealing to children	Coupons, contests or giveaways to be entered or redeemed later that are not specifically appealing to children.	Tote bags Access to a free weight loss plan Patio furniture set
