# Supplementary material for: Quantifying Child-Appeal: The Development and Mixed-Methods Validation of a Methodology for Evaluating Child-Appealing Marketing on Product Packaging
Source: Int J Environ Res Public Health. 2021 Apr 29;18(9):4769. doi: 10.3390/ijerph18094769 (PMC8124606; doi:10.3390/ijerph18094769)
Supplement: Supplementary file 1 [file ijerph-18-04769-s001.zip › Supplementary Table 1 (IJERPH).pdf]

Supplementary Table 1. Mock cereal boxes used in child-appealing packaging (CAP) coding tool validation study

Box	Front & Back (if not identical)	Child-appealing packaging? (Yes/No)	Core and Broad marketing techniques displayed	Marketing Power Score
**A**	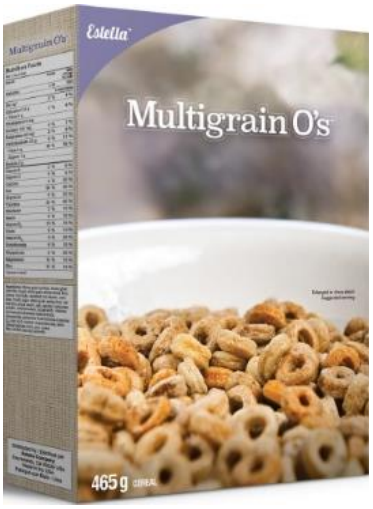	No	**Core:**<br>- N/A<br><br>**Broad:**<br>- N/A	0
**B**	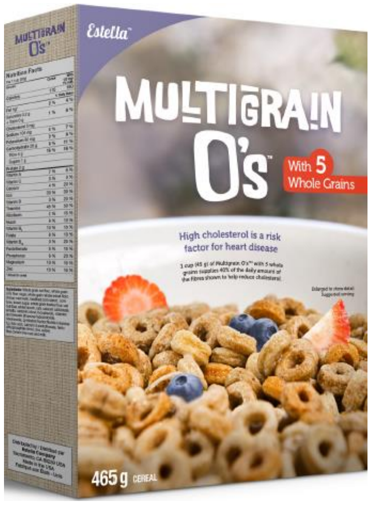	No	**Core:**<br>- N/A<br><br>**Broad:**<br>- Appeals to health<br>- Fun or child-appealing lettering	2

Box	Front & Back (if not identical)	Child-appealing packaging? (Yes/No)	Core and Broad marketing techniques displayed	Marketing Power Score
C	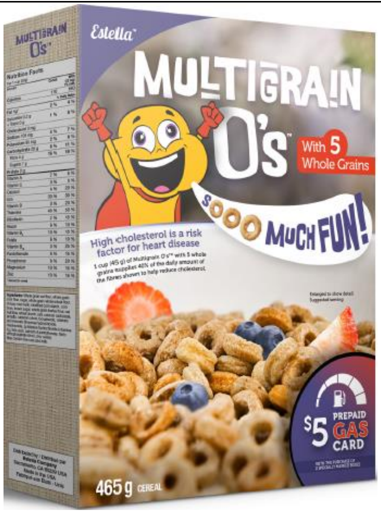	Yes	**Core:**<br>- Presence of branded characters<br>- Appeals to fun<br><br>**Broad:**<br>- Appeals to health<br>- Fun or child-appealing lettering<br>- Coupons, contests, or giveaways, not specifically appealing to children	5
D	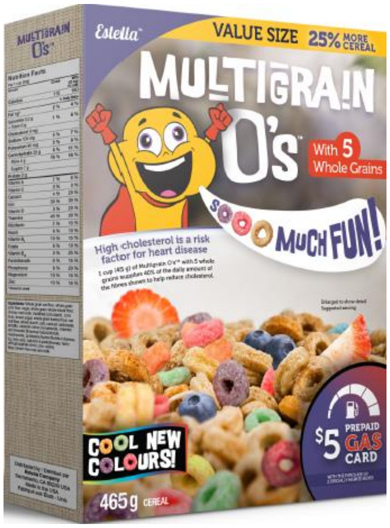	Yes	**Core:**<br>- Presence of branded characters<br>- Appeals to fun<br>- Unconventional colour of the product, featured on the package<br>- Appeals to coolness or novelty<br><br>**Broad:**<br>- Appeals to health<br>- Fun or child-appealing lettering<br>- Coupons, contests, or giveaways, not specifically appealing to children<br>- Appeals to other product benefits (e.g., value)	8

Box	Front & Back (if not identical)	Child-appealing packaging? (Yes/No)	Core and Broad marketing techniques displayed	Marketing Power Score
E	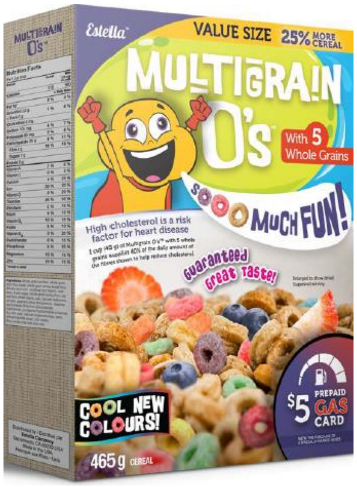<br>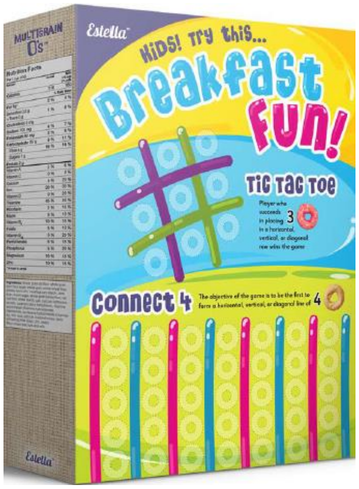	Yes	**Core:**<br>- Presence of branded characters<br>- Appeals to fun<br>- Unconventional colour of the product, featured on the package<br>- Appeals to coolness or novelty<br>- Presence of games or activities on package<br>- Child-appealing visual/graphical design of package<br><br>**Broad:**<br>- Appeals to health<br>- Fun or child-appealing lettering<br>- Coupons, contests, or giveaways, not specifically appealing to children<br>- Appeals to other product benefits (e.g., value)<br>- Appeals to taste	11

<table>
<tr><th>Box</th><th>Front & Back (if not identical)</th><th>Child-appealing packaging? (Yes/No)</th><th>Core and Broad marketing techniques displayed</th><th>Marketing Power Score</th></tr>
<tr>
<td>F</td>
<td>
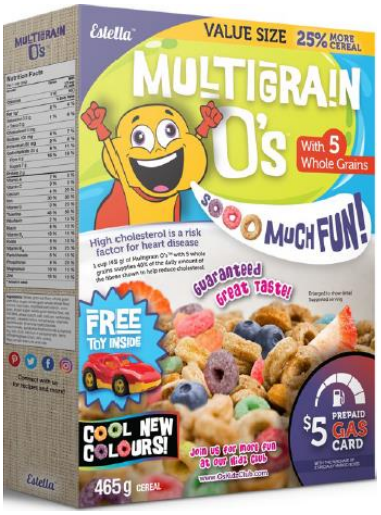

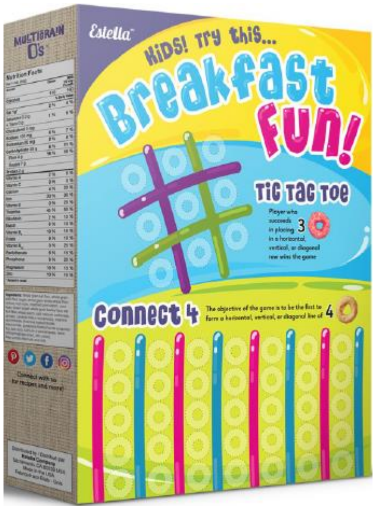

</td>
<td>Yes</td>
<td>
Core:

Presence of branded characters
Appeals to fun
Unconventional colour of the product, featured on the package
Appeals to coolness or novelty
Presence of games or activities on package
Child-appealing visual/graphical design of package
Coupons, contests, or giveaways, specifically appealing to children
Promotion of websites, social media, rewards programs, specifically appealing to children

Broad:

Appeals to health
Fun or child-appealing lettering
Coupons, contests or giveaways, not specifically appealing to children
Appeals to other product benefits (e.g., value)
Appeals to taste
Promotion of websites, social media, rewards programs, not specifically appealing to children

</td>
<td>14</td>
</tr>
</table>
